# Immunochemical Identification of the Main Cell Wall Polysaccharides of the Early Land Plant *Marchantia polymorpha*

**DOI:** 10.3390/cells12141833

**Published:** 2023-07-12

**Authors:** Hasan Kolkas, Vincent Burlat, Elisabeth Jamet

**Affiliations:** Laboratoire de Recherche en Sciences Végétales, Université de Toulouse, CNRS, UPS, Toulouse INP, Auzeville-Tolosane, France; hasan.kolkas94@gmail.com (H.K.); vincent.burlat@univ-tlse3.fr (V.B.)

**Keywords:** cell wall, *Marchantia polymorpha*, polysaccharides, immunolocalization

## Abstract

Plant primary cell walls are composite structures surrounding the protoplast and containing pectins, hemicelluloses, and cellulose polysaccharides, as well as proteins. Their composition changed during the evolution of the green lineage from algae to terrestrial plants, i.e., from an aquatic to a terrestrial environment. The constraints of life in terrestrial environments have generated new requirements for the organisms, necessitating adaptations, such as cell wall modifications. We have studied the cell wall polysaccharide composition of thalli of *Marchantia polymorpha*, a bryophyte belonging to one of the first land plant genera. Using a collection of specific antibodies raised against different cell wall polysaccharide epitopes, we were able to identify in polysaccharide-enriched fractions: pectins, including low-methylesterified homogalacturonans; rhamnogalacturonan I with arabinan side-chains; and hemicelluloses, such as xyloglucans with XXLG and XXXG modules, mannans, including galactomannans, and xylans. We could also show the even distribution of XXLG xyloglucans and galactomannans in the cell walls of thalli by immunocytochemistry. These results are discussed with regard to the cell wall proteome composition and in the context of the evolution of the green lineage. The cell wall polysaccharides of *M. polymorpha* illustrate the transition from the charophyte ancestors of terrestrial plants containing xyloglucans, xylans and mannans as hemicelluloses, and embryophytes which do not exhibit mannans as major primary cell wall polysaccharides.

## 1. Introduction

*Marchantia polymorpha* has recently become an attractive model, as it is an early land plant belonging to the bryophytes, which include terrestrial plants without vascular systems, like mosses, liverworts, and hornworts [1]. The study of such plants should contribute to a better understanding of the evolution of the green lineage, especially the transition from aquatic to terrestrial environments about 450 MYA [2,3]. *M. polymorpha* can be easily grown in laboratory conditions, it has a short life cycle, and the dominant phase is the haploid gametophyte [3]. It has a small genome comprising around 20 thousand predicted genes [4]. In addition, it can be easily transformed using *Agrobacterium tumefaciens*, and CRISPR-Cas9 gene editing allows targeted mutations [2].

The composition and structure of plant cell walls are highly variable, depending on the cell wall micro-domains, the organs/tissues, the stage of development, the plant species, and environmental cues [5,6,7,8,9]. Primary cell walls allowing growth are mainly composed of pectins, hemicelluloses, cellulose, and proteins [10,11]. Pectins are a family of molecules comprising homogalacturonans (HGs), which can be methylesterified and acetylated, and rhamnogalacturonans I (RG-I) and II (RG-II) [12]. Hemicelluloses are also diverse and comprise xyloglucans (XGs), which are the major hemicelluloses of dicot plants, mannans, which are more abundant in early land plants, and xylans, found in the primary walls of monocot plants and in secondary walls of flowering plants [13]. At the end of growth, secondary walls are synthesized, among which are those containing phenolic or lipidic compounds, e.g., lignified walls that form wood [14], and walls rich in cutin and waxes that form the cuticle at the surface of aerial organs [15].

The transition of plants from the aquatic to the terrestrial environment about 450 MYA has required numerous adaptations of the cell walls, e.g., (i) tighter regulation of water exchange to limit desiccation, enabled by the presence of hydrophilic hemicelluloses, like mannans [16], and the acquisition of a cuticle barrier [17]; (ii) protection against UV radiation through cell wall-embedded phenolic compounds, as well as vacuolar phenolics [18] and the cuticle [19]; or (iii) the capacity to synthesize lignified secondary walls to allow vertical growth and water transport in tracheophytes [20]. To better understand this transition and the subsequent evolution of land plants, many studies have been devoted to the analysis of the structure and composition of the cell wall in the green lineage [19,20,21,22,23,24]. For example, mannans are the main hemicelluloses in charophytes and were found in several bryophytes by paper chromatography after acid hydrolysis of the cell walls [25], but they have been replaced by other hemicelluloses in spermatophytes [13]. The XGs of mosses and liverworts were shown by mass spectrometry analyses to be structurally distinct from those of flowering plants and to contain galacturonic acid [26]. The cell walls of mosses and liverworts were also found to contain p-coumaric and ferulic acids [18]. Regarding the cell wall proteins, we have recently provided a deep analysis of the cell wall proteome of *M. polymorpha* thalli and identified 410 different proteins, corresponding to about one third of the predicted cell wall proteome [27]. In addition, arabinogalactan proteins have been characterized in *M. polymorpha* and exhibit specific features, like the presence of terminal 3-O-methyl-rhamnose in their glycan moieties, and highly branched galactan side-chains that contain only traces of β-1,6-linked galactose, unlike angiosperms [28,29]. 

In this work, we took advantage of the large collection of monoclonal antibodies raised against specific cell wall epitopes to probe cell wall polysaccharides in *M. polymorpha* thalli. These results complement the present knowledge on bryophyte cell wall composition, and support our previous analyses suggesting that the cell wall proteome composition is tightly related to the polysaccharide composition. In addition, the polysaccharide content of *M. polymorpha* cell walls is discussed with regard to the particular position of this plant in the evolution of the green lineage at the transition between charophytes and embryophytes.

## 2. Materials and Methods

### 2.1. Plant Material and Growth Conditions

*M. polymorpha* thalli of male accession Takaragaike-1 (Tak-1) were maintained asexually from single gemmae. They were grown for 2 weeks on half-strength Gamborg’s B-5 basal medium (GB1/2), with minimal organics medium (Sigma-Aldrich, Merck, Darmstadt, Germany), supplemented with 1% sucrose and 1.4% agar at pH 5.5. The 3- and 4-week-old thalli were grown for 2 weeks in vitro, and then for 1 or 2 weeks on Jiffy pellets (Jiffy Products International AS, Stange, Norway). They were placed in a growth chamber at 22 °C under 100 µE m^−2^.s^−1^ light intensity and an 8 h dark/16 h light photoperiod. Only the apical parts of the thalli, i.e., the actively growing tissues, were used.

### 2.2. Extraction of Cell Wall Polysaccharide Fractions

For each of the 3 biological replications, 30 g of thalli were lyophilized and subsequently ground into a fine powder in liquid nitrogen, using a mortar and a pestle, to obtain 1 g of starting material. The protocol for the sequential extraction of pectins and hemicelluloses was as described [28]. Briefly, the first step consisted in the removal of phenolic compounds through two successive incubations in a 70% acetone solution: 1 g powder/10 mL acetone, then 1 g/100 mL. The pellet was then dried prior to the sequential extraction of the polysaccharides, using 1 mL of each of the following solutions: 0.2 M (NH_4_)_2_C_2_O_4_ to get a first pectin-enriched fraction (P1); 3% Na_2_CO_3_ (m/v) to get a second demethylesterified pectin-enriched fraction (P2); and 2 M KOH to get a hemicellulose-enriched fraction (H). A final step was added in order to obtain a cellulose-enriched fraction (C), using a cadoxen solution (0.78 M CdO in 31% *v*/*v* 1,2 diaminoethane) (m/v, 1/10) [30]. Each extraction step was performed at 70 °C, for 21 h, using a rotating wheel. The supernatants, corresponding to the enriched fractions, were collected after each step by centrifugation at 17,000× *g* for 10 min. All the extracts were stored at 4 °C until use. 

### 2.3. Polysaccharide Arrays

The polysaccharide array protocol was designed in accordance with [31]. The polysaccharide extracts were diluted in water (*v*/*v*, 1/25) and 40 µL of these dilutions was loaded into each well of a Bio-Dot^®^ apparatus (BIO-RAD, Marnes-la-Coquette, France), onto a 0.45 µm nitrocellulose blotting membrane (AmershamTM ProtranTM, Dutscher, Bernolsheim, France). The membrane was then blocked with 5% BSA in TBS-T (20 mM Tris-HCl, pH 7.5, 150 mM NaCl, 0.05% (m/v) Tween 20) for 1 h at room temperature. It was then incubated overnight at 4 °C, in the presence of a 1/250 (*v*/*v*) dilution of primary monoclonal antibodies in TBS-T and 1% BSA. 

All the antibodies were from Kerafast (Boston, MA, USA), with the exception of RU1 and RU2, which were kindly provided by Dr M.-C. Ralet (INRAe Nantes, France) [32] (Table 1).

After three washes with TBS-T, the membrane was incubated for 2 h at room temperature in the presence of two types of IgG secondary antibodies, each conjugated to alkaline phosphatase (Sigma-Aldrich), diluted 1/10,000 in TBS-T and 1% BSA: (i) an anti-rat IgG secondary antibody for the rat JIM and LM primary antibodies or (ii) an anti-mouse IgG secondary antibody for the mouse RU1 and RU2 primary antibodies. After three washes with TBS-T, the activity of the alkaline phosphatase was detected after incubation in the presence of a mixture of 33 µL of BCIP (5-bromo-4-chloro-3-indolyl phosphate, 50 mg/mL) and 33 µL NBT (nitro-blue tetrazolium chloride, 50 mg/mL) in 10 mL TBS, as substrates (Sigma-Aldrich).

The polysaccharides used as positive controls were ordered from either Sigma-Aldrich: citrus low-methylesterified HG (ref 3850), apple high-methylesterified HG (ref 76282), and beechwood xylan (ref X4252); or from Megazyme (Bray, Ireland): potato RG-I (P-RHAM1), sugarbeet arabinan (P-ARAB), tamarin XG (P-XYGLN), and ivory nut mannan (P-MANIV). Twenty µg of each polysaccharide aqueous solution was deposited onto the nitrocellulose membrane. 

### 2.4. Immunocytochemistry of Cell Wall Epitopes

The apical parts of the thalli were taken after 2, 3 or 4 weeks of growth. The fixation of the samples was performed as previously described [34]. Briefly, the first step was a vacuum-infiltration in a solution of formaldehyde acetic acid ethyl alcohol (FAA) containing 10% formalin (3.7% final concentration of formaldehyde), 5% acetic acid, and 50% ethanol in water. The samples were then washed and stored at 4 °C in 50% ethanol. The next step was a progressive infiltration at 30 °C with an ethanol/tert-butanol series, from 40% ethanol/10% tert-butanol to 100% tert-butanol. They were then progressively infiltrated with paraffin (Paraplast Plus^®^, Sigma-Aldrich) following a step-by-step procedure performed at 60 °C, from 50% tert-butanol/50% paraffin, to 100% paraffin. The thalli were individually embedded in paraffin in individual molds. The samples were assembled in tissue arrays allowing simultaneous cross sectioning. Serial sections of 12 µm thickness were cut with a microtome and deposited onto slides pre-coated with silane. The paraffin was finally removed with 100% xylene (twice for 15 min), followed by an incubation in 100% ethanol (twice for 5 min). The sections were progressively rehydrated in decreasing concentrations of ethanol. The sections were blocked for 30 min in 5% non-fat dry milk diluted in TTBS (10 mM Tris-HCl pH 7.5, 500 mM NaCl, 0.3% (w/v) Triton X-100). The labeling of the cell wall epitopes was performed with the monoclonal antibodies diluted 10 times in TTBS-milk and incubated for 3 h at room temperature under a coverslip. After washing with TTBS (6 times for 5 min), the slides were incubated for 90 min with the secondary antibody conjugated with alkaline phosphatase and diluted 1/100. Finally, the detection of alkaline phosphatase activity was performed as with the polysaccharide arrays (see Section 2.3). The samples were finally mounted under a coverslip in three drops of Eukitt^®^ mounting medium (Freiburg, Germany) and were scanned with a 20× objective lens (0.46 µm/pixel), with a Nanozoomer 2.0HT (Hamamatsu, Massy, France) in the brightfield mode. The images were visualized with the NDP.view software (Hamamatsu, version NDP.view2).

## 3. Results

### 3.1. Low-Methylesterified Homogalacturonans Are Major Pectins in M. polymorpha Thalli Cell Walls

**Figure 1:**
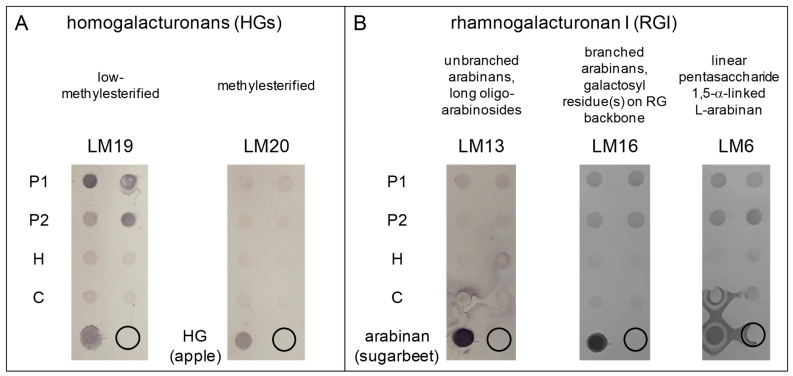
Polysaccharide arrays showing that low-methylesterified homogalacturonans are the major pectins in *M. polymorpha* thalli cell walls. Different polysaccharide fractions have been sequentially extracted from the cell walls of 3-week-old *M. polymorpha* thalli. The polysaccharide arrays have been probed with monoclonal antibodies specific for different cell wall pectin epitopes: (**A**) LM19 and LM20 detect low- and partially-methylesterified homogalacturonans, respectively; (**B**) LM13, LM16, and LM6 detect different parts of RG I as indicated on the figure. For each antibody, one representative experiment with two technical replicates is shown, out of three biological replications. P1 and P2 correspond to pectin-enriched fractions; H, to the hemicellulose-enriched fraction; and C, to the cellulose-enriched fraction (see Section 2 for details). For each probed polysaccharide, a relevant positive control is included on the array, as indicated. The circles represent the background level with no polysaccharide deposited on the membrane.

Two different pectin-enriched fractions have been successively extracted from the cell walls of *M. polymorpha* 3-week-old thalli [35]. As a calcium-chelating agent, (NH_4_)_2_C_2_O_4_ allowed the extraction of low-methylesterified and weakly-bound pectins, including those present in the middle lamella (P1 fraction). The Na_2_CO_3_ treatment allowed the extraction of covalently-bound pectins and the demethylesterification of pectins (P2 fraction). The polysaccharide arrays were then probed with the monoclonal antibodies LM19 and LM20, which are specific for low- and partially-methylesterified HGs, respectively (Figure 1A).

In the same way, we used the monoclonal antibodies RU1 and RU2, which are both specific for the RG-I backbone, and LM13, LM16, and LM6, which are specific for different side-chains of the RG-I molecules (Figure 1B and Figure S1). In all cases, the negative control dots, with no polysaccharides deposited, showed no signal. The positive controls, corresponding to low-methylesterified (from citrus) and high-methylesterified (from apple) HGs, or arabinan (RG-I branching) from sugarbeet, showed strong positive signals. The positive control for RU2, i.e., RG-I from potato, showed no signal (Figure S1). We assume that its structure is different from that of the sugarbeet RG-I used to obtain the RU2 antibody [36].

LM19 revealed strong signals in the P1 and P2 pectin-enriched fractions, whereas LM20 only showed weak signals in all the fractions (Figure 1A). Weak signals were observed with the RU1 and RU2 antibodies in P1, P2, and in the cellulose-enriched fraction (C), whereas a very weak signal was observed in the hemicellulose-enriched fraction (H) (Figure S1). Since a previous study on the monosaccharide composition of *M. polymorpha* cell walls has suggested the presence of a rhamnogalacturonan backbone [37], we assume that the observed signals are relevant, even if weak. Signals were also observed in P1 with LM13, and in P1 and P2 with LM16 and LM6, consistent with previous observations regarding the presence of rhamnogalacturonan in *M. polymorpha* cell walls [37] (Figure 1B).

These results indicate that low-methylesterified HGs are major pectic polysaccharides in *M. polymorpha* cell walls compared to partially-methylesterified HGs, and that RG-I with linear and branched arabinans are also present.

### 3.2. Xyloglucans and Mannans Are Present in All the Cell Walls of M. polymorpha Thalli

**Figure 2:**
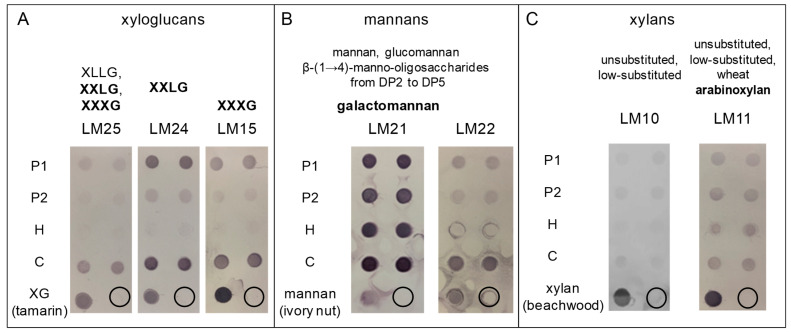
Polysaccharide arrays showing the presence of xyloglucans, mannans, and xylan in *M. polymorpha* thalli cell walls. Different polysaccharide fractions have been sequentially extracted from the cell walls of 3-week-old *M. polymorpha* thalli. The polysaccharide arrays have been probed with monoclonal antibodies specific for different cell wall hemicellulose epitopes: (**A**) LM25, LM24, and LM15 detect xyloglucans with different modules, XLLG, XXLG, and XXXG, respectively; (**B**) LM21 and LM22 detect different kinds of mannans, as indicated on the figure; (**C**) LM10 and LM11 detect different types of xylans, with LM11 recognizing wheat arabinoxylan. For each antibody, one representative experiment with two technical replicates is shown, out of three biological replications. P1 and P2 correspond to pectin-enriched fractions; H, to the hemicellulose-enriched fraction; and C, to the cellulose-enriched fraction (see Section 2 for details). For each probed polysaccharide, a relevant positive control is included on the array as indicated. The circles represent the background level with no polysaccharide deposited on the membrane.

After the extraction of pectins, a hemicellulose-enriched fraction (H) and a cellulose-enriched fraction (C) were sequentially obtained using 2 M KOH and cadoxen, respectively. Based on previous work focused on monosaccharide analyses of *M. polymorpha* cell walls showing the presence of significant amounts of arabinose, xylose, mannose, glucose, and galactose [28], we focused our study on XGs, mannans, and xylans. LM25, LM24, and LM15 were used to probe XGs, LM21 and LM22 to probe mannans, with LM21 recognizing galactomannans, and LM10 and LM11 to probe xylans. Note that the antibodies recognized the polysaccharides used as respective positive controls in all cases (Figure 2).

XGs are β-1,4 glucans which can be substituted by diverse monosaccharide residues [33]. The structures of the different modules recognized by LM25, LM24, and LM15 are detailed in Table 1. 

LM25, which recognizes the XLLG, XXLG, and XXXG modules of XGs, revealed a weak signal in the P1 fraction, but a strong one in the C fraction (Figure 2A). This result suggested a strong association of the XGs recognized by LM25 with cellulose microfibrils. On the contrary, both LM24 and LM15 revealed strong signals in the P1 and C fractions (Figure 2A). This result showed that XGs with XXLG and XXXG modules were present in the cell walls of *M. polymorpha*. In addition, these XGs were distributed in two different fractions: (i) either labile or associated with weakly bound pectins, as suggested by their presence in P1; (ii) or strongly associated with cellulose microfibrils, as suggested by their presence in C. Surprisingly, no XGs were found in the hemicellulose-enriched fraction (H) with these three antibodies. This latter feature illustrates the fact that the sequential extraction of cell wall polysaccharides can provide information on the relative lability/association with cell wall polymers.

Regarding mannans, LM21, which recognized a large spectrum of epitopes including galactomannans, revealed strong signals in all the cell wall fractions, in contrast with LM22, which only revealed weak signals in P1 and C (Figure 2B). These results suggested that: (i) galactomannans are highly abundant in *M. polymorpha* cell walls since they were detected in all the fractions, from the more labile (P1), to those strongly bound to cellulose microfibrills (C); (ii) there are other types of mannans, either glucomannans or short mano-oligosaccharides, recognized by LM22, but not by LM21, which were not found in fraction H.

Finally, the LM11 and LM10 antibodies revealed the presence of xylans, but the signals were weaker with LM10 than with LM11, which recognizes wheat arabinoxylan (Figure 2C).

### 3.3. Mannans and XXLG Xyloglucan Epitopes Are Accessible in All the Cell Walls of M. polymorpha Thalli

Based on the results obtained with the polysaccharide arrays, we chose three antibodies which gave strong signals to look at the distribution of the corresponding epitopes in *M. polymorpha* thalli at three different stages of development (2, 3, and 4 weeks after sub-culturing gemmae on fresh medium in vitro): LM19 for low-methylesterified HGs, LM24 for XXLG modules of XGs, and LM21 for mannan, glucomannan, and galactomannan.

**Figure 3:**
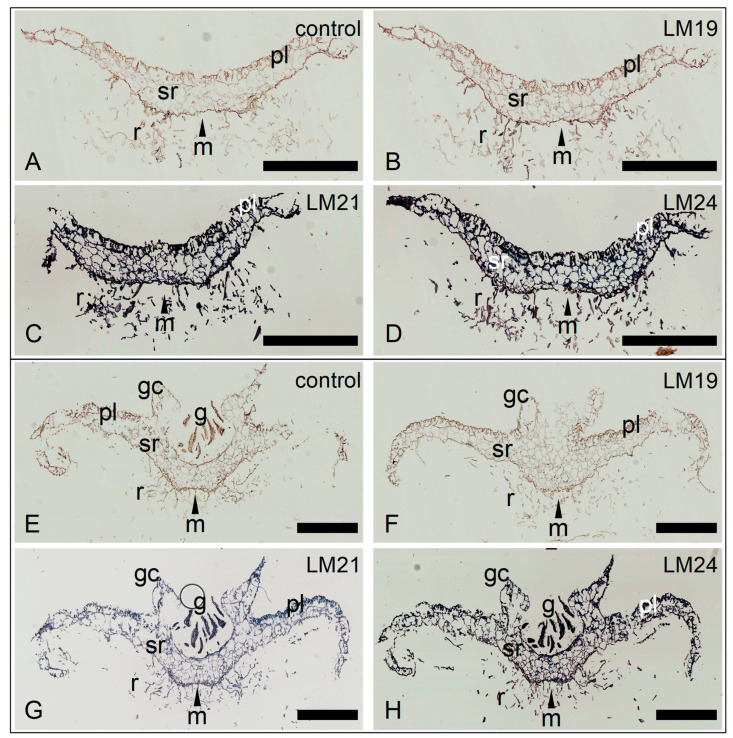
Immunocytochemical labeling of low-methylesterified homogalacturonans, mannans, and xyloglucans in the cell walls of *M. polymorpha* thalli. Two stages of development have been analyzed: (**A**–**D**) 2-week-old thalli; (**E**–**H**) 4-week-old thalli. Paraffin-embedded serial sections have been probed with monoclonal antibodies specific for different cell wall epitopes: (**A**,**E**) control with no primary antibody; (**B**,**F**) LM19, recognizes low-methylesterified homogalacturonans; (**C**,**G**) LM21, recognizes different kinds of mannans, including galactomannans; (**D**,**H**) LM24, recognizes xyloglucans with XLLG modules. g: gemmae; gc: gemma cup; m: midrib, pl: photosynthetic layer; r: rhizoid; sr: storage region. Scale bars = 1 mm.

At the three developmental stages, the control without primary antibody gave no signal (Figure 3A,E and Figure S2A). Surprisingly, we observed no signal with the LM19 antibody, suggesting that despite their apparent lability as observed on polysaccharide arrays, the corresponding epitopes were not accessible on thalli sections (Figure 3B,F and Figure S2B). On the contrary, all the cell walls, including those of rhizoids, gemma cups, and gemmae, were labeled with the LM21 and LM24 antibodies, consistent with the results obtained with the polysaccharide arrays (Figure 3C,D,G,H and Figure S2C,D).

## 4. Discussion

This work provides new information on the composition of the cell walls of *M. polymorpha* thalli, including rhizoids. Using polysaccharide arrays, it has been possible to show the presence of low-methylesterified HGs, RG-I with arabinan side-chains, XGs with XXLG and XXXG modules, and mannans, including galactomannans. In addition, immunocytochemical labeling has shown that some polysaccharides (XGs with XXLG modules and galactomannans) are present in all the cell walls of thalli at three different developmental stages, from 2 to 4 weeks after gemmae sub-culturing.

The presence of HGs was consistent with that of uronic acids in acidic hydrolysates of cell wall fractions from *M. polymorpha* thalli or cell suspension cultures [28,37]. We could show that low-methylesterified HGs were major HGs compared to partially-methylesterified HGs, and that it was not possible to detect low-methylesterified HG epitopes on tissue sections. We assume that they were not accessible. In a previous study, low- and partially-methylesterified HGs could be detected with both JIM5 and JIM7 antibodies, respectively, on tissue sections of several leafy liverworts, with a focus on water-conducting cells [38]. The detection of high levels of LM19 epitopes on polysaccharide arrays is consistent with the identification of six pectin methylesterases (PMEs) in the *M. polymorpha* thalli cell wall proteome and the absence of predicted PME inhibitors in the *M. polymorpha* genome [27]. In addition, two polysaccharide lyases and two polygalacturonases (glycoside hydrolase family 28) were identified in the cell wall proteome. The low-methylesterified HGs can form the so-called egg-boxes, with calcium ions stiffening the cell wall [39]. They can also be substrates for either polygalacturonases or lyases, thus releasing oligogalacturonides, which could be signal molecules or contribute to cell wall loosening. Changes in the extracellular pH could influence the balance between these two types of enzymatic activities, with a higher pH favoring the enzymatic activity of PMEs, and a lower pH favoring acylesterases and lyases [39]. Surprisingly, it has not been possible to map the HG epitopes on thalli sections with LM19, specific for low-methylesterified HGs. We assume that they were masked by the other cell wall components. 

The presence of RG-I was detected with different antibodies recognizing either the RG-I backbone or the arabinan side-chains. This result is consistent with the presence of rhamnose in acidic hydrolysates of *M. polymorpha* cell wall fractions [28,37]. In particular, short and long linear arabinoside side-chains were detected with the LM6 and LM13 antibodies, respectively. RG-I side-chains have been assumed to play roles in the resistance of plants to desiccation, by preserving cell wall flexibility and thereby allowing recovery of the tissues after rewatering [36]. This property could have played a role in the adaptation to the terrestrial environment. HGs and pectins with a rhamnogalacturonan backbone have been estimated to account for 67% and 10% of the pectic polysaccharides in *M. polymorpha* cell cultures, respectively [37]; whereas HGs and rhamnogalacturonans (I and II) were estimated to be present in similar proportions in *Arabidopsis thaliana* leaves [40]. In addition, in guard cells of *A. thaliana*, the arabinan RG-I side-chains would play a role in cell wall flexibility, by preventing the formation of egg-box structures and thus acting as regulators of polysaccharide proximity [41].

Different types of hemicelluloses have been detected, such as XGs, mannans, and xylans. XGs and mannans could also be detected by immunolocalization in all the cell walls of thalli, including rhizoids, at three stages of development from 2 to 4 weeks of growth after gemmae sub-culturing. These results were consistent with the presence of xylose and mannose in acidic hydrolysates of *M. polymorpha* cell wall fractions [28]. XGs and mannose were also detected after enzymatic or acidic digestion in the walls of *Lunularia cruciata*, a thalloid liverwort, and of several leafy liverworts [25]. Moreover, XGs were detected with LM25 in a liquid medium of *M. polymorpha* [42]. Mannose-containing hemicelluloses and XGs were found in the walls of charophytes, which are assumed to be the closest ancestors to land plants [25,43]. However, a previous work using mass spectrometry and nuclear magnetic resonance analyses of purified XG fractions concluded there was a predominance of XXGG modules in *M. polymorpha* [26], e.g., due to the presence of a xylose side-chain (X pattern) or the absence of a side-chain (G pattern) (see [33] for the nomenclature of XG side chains). In our study, we also found XXXG and XXLG (the L pattern corresponds to the presence of a xylose-galactose side chain) modules. Both studies agree on the presence of X and L side-chains. The discrepancy could be explained by the fact that the signals revealed by the antibodies were found in our P1 pectin- and hemicellulose-enriched fractions, which are very different from a purified XG fraction. Different types of XGs with different kinds of interactions with the other cell wall polysaccharides could be present in *M. polymorpha*. Conversely, the antibodies which were used in this study did not recognize XXGG modules and no signal was revealed in the hemicellulose-enriched fraction with the LM25, LM24, and LM15 antibodies. The presence of XGs can be correlated with that of glycoside hydrolases of the GH16 family, comprising xyloglucan endotransglycosylases/hydrolases (XTHs), in the cell wall proteome of *M. polymorpha* [27]. Similarly, three glycoside hydrolases of the GH5 family predicted to have a trans-β-mannanase or a mannan endo-β-1,4-mannosidase activity [6] were identified in this proteome. In addition, several lectins with a predicted mannose-binding domain were also found [27]. Since the signals obtained with LM11 were stronger than those obtained with LM10, we can suppose that the structure of the *M. polymorpha* xylans is close to that of wheat arabinoxylans, i.e., substituted xylans [13]. This result is different from that of a previous work where no labeling could be found on tissue sections of different liverworts and moss tissues using the same antibodies [44]. However, in that study *M. polymorpha* was not included and only the thallus nerve and the gametophyte were analyzed, whereas we looked at whole thalli. As with XGs and mannans, xylans were previously detected in the cell walls of charophytes [43]. Xylans are also major polysaccharides of the primary cell walls of monocot plants [10]. 

## 5. Conclusions

Altogether, this work complements previous work devoted to the identification of the cell wall monosaccharides and polysaccharides present in bryophytes. In *M. polymorpha*, the major polysaccharides of the primary cell walls of flowering plants are present, i.e., HGs, RG-I, XGs, and xylans, together with mannans which are also present in charophytes, the most probable ancestors of land plants. Mannan epitopes are particularly abundant and could play roles in the resistance to desiccation which has been a new challenge for land plants. Regarding XGs and xylans, they are found in the primary cell walls of monocot and dicot plants, respectively [10]. Interestingly, we showed the presence of XGs with XXLG and XXXG modules in unexpected cell wall fractions, i.e., in the pectin-enriched fraction where they could be loosely associated with the other cell wall polymers, and in the cellulose-enriched fraction where, on the contrary, they could be strongly associated with cellulose microfibrils. We also showed a homogeneous distribution (or masking) of the studied epitopes in all of the thallus cell types at the three studied developmental stages. It suggests a relatively simple homogeneous cell type organization of *M. polymorpha* cell walls that is different from the complex cell-specific and even microdomain-specific cell wall organization encountered in tracheophytes [5]. *M. polymorpha* cell walls thus nicely illustrate the evolution from charophytes to embryophytes, also corresponding to the transition from an aquatic to a terrestrial environment, and the later evolution towards flowering plants. 

## Figures and Tables

**Table 1:** Specificities of the monoclonal antibodies against different cell wall epitopes.

Cell Wall Polysaccharides	Monoclonal Antibody	Specificity
pectins	LM19	low-esterified homogalacturonans
	LM20	high-methylesterified homogalacturonans
	LM13	unbranched arabinans, long oligo-arabinosides
	LM16	branched arabinans, galactosyl residue(s) on RG backbones
	LM6	linear pentasaccharide of 1,5-α-linked L-arabinan epitopes
	RU1	[→2)-α-L-rhamnose p-(1→4)-α-D-galacturonic acid p-(1→]_7_, at least 6 disaccharide «rhamnose-galacturonic acid»
	RU2	[→2)-α-L-rhamnose p-(1→4)-α-D-galacturonic acid p-(1→]_7_, at least 2 disaccharide «rhamnose-galacturonic acid» repeats, tolerates galactose substitutions
hemicelluloses *	LM25	xyloglucans (XLLG, XXLG, XXXG modules) *
	LM24	xyloglucans (XXLG module) *
	LM15	xyloglucans (XXXG module) *
	LM21	mannan, glucomannan, galactomannan, β-(1→4)-manno-oligosaccharides from DP2 to DP5
	LM22	mannan, glucomannan, β-(1→4)-manno-oligosaccharides from DP2 to DP5
	LM10	unsubstituted and relatively low-substituted xylans
	LM11	unsubstituted and relatively low-substituted xylans, wheat arabinoxylan

* The nomenclature of XG modules is described in [33]. Side chains are named as follows: G stands for no side-chain; L for galactose-xylose side-chains (β-D-Gal-(1→2)-α-D-Xyl-(1→6)-β-D-Glc linkage); and X for xylose side-chains (α-D-Xyl-(1→6)-β-D-Glc linkage).

## Data Availability

All the data are included in the article.

## Supplementary Materials

The following supporting information can be downloaded at: https://www.mdpi.com/article/10.3390/cells12141833/s1, Figure S1: Polysaccharide arrays showing the presence of rhamnogalacturonan I (RG-I) in *M. polymorpha* thalli cell walls; Figure S2: Immunolocalization of low-methylesterified homogalacturonans, mannans, and xyloglucans in the cell walls of 3-week-old *M. polymorpha* thalli.
