# Supplementary material for: Immunochemical Identification of the Main Cell Wall Polysaccharides of the Early Land Plant Marchantia polymorpha
Source: Cells. 2023 Jul 12;12(14):1833. doi: 10.3390/cells12141833 (PMC10378070; doi:10.3390/cells12141833)
Supplement: Supplementary file 1 [file cells-12-01833-s001.zip › cells-2459076-supplementary.pdf]

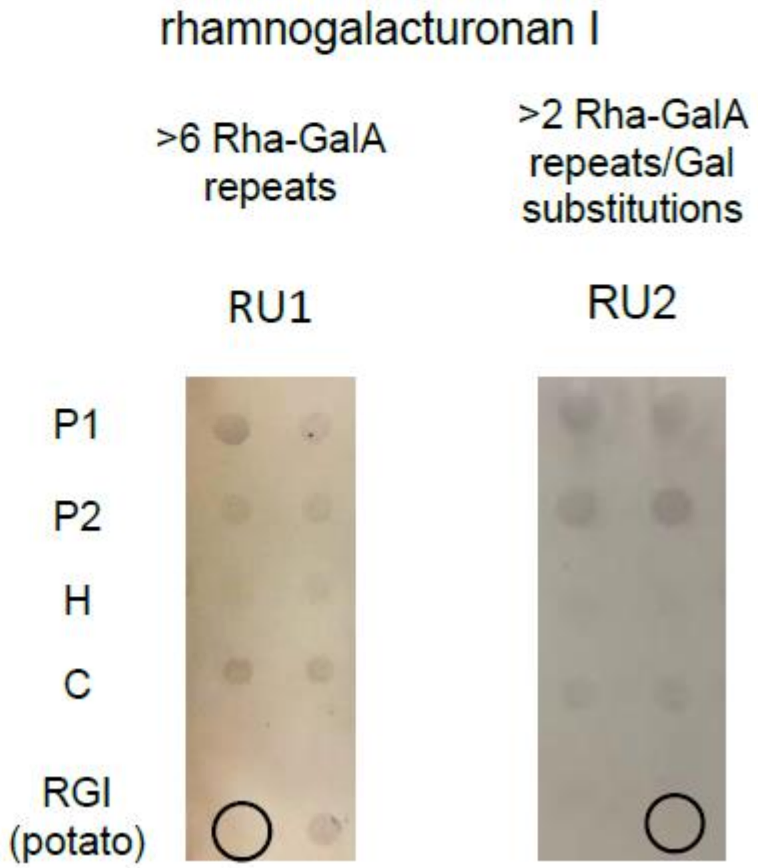

Figure S1. Polysaccharide arrays showing the presence of rhamnogalacturonan I (RGI) in M. polymorpha thalli cell walls. Different polysaccharide fractions have been extracted from cell walls of 3 week-old M. polymorpha thalli. The polysaccharide arrays have been probed with monoclonal antibodies specific for different cell wall pectin epitopes: RU1 and RU2 detect RGI. Note that the positive control did not give any signal with RU2. For each antibody, one representative experiment with two technical replicates is shown out of three biological replicates. P1 and P2 correspond to pectin-enriched fractions, H, to the hemicelluloses-enriched fraction, and C, to the cellulose-enriched fraction (see section 2 for details). The circle represents the background level with no polysaccharide deposited on the membrane.

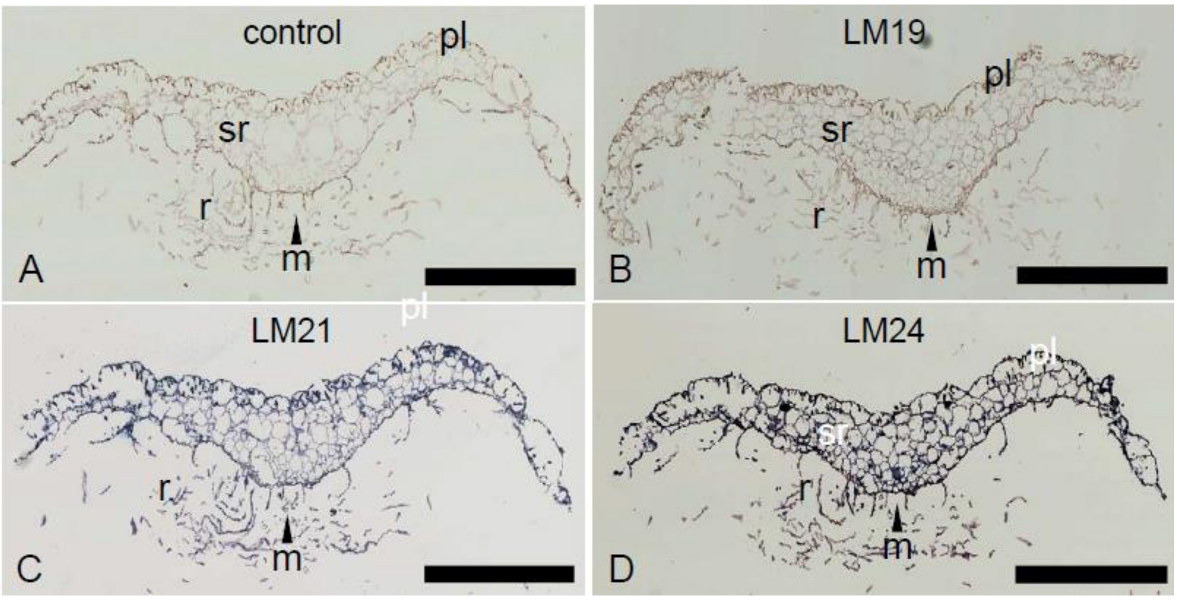

Figure S2. Immunocalization of low methylesterified homogalacturonans, mannans and xyloglucans in the cell walls of 3 week-old *M. polymorpha* thalli. Thin sections have been probed with monoclonal antibodies specific for different cell wall epitopes: **A**) control with no primary antibody; **B**) LM19 recognizes low methylesterified homogalacturonans; **C**) LM21 recognizes different kinds of mannans, including galactomannans; **D**) LM24, recognizes xyloglucans with XLLG lateral chains; m: midrib, pl: photosynthetic layer; r: rhizoid; sr: storage region. Scale bars= 1 mm.
